# Fluoxetine Promotes Hippocampal Oligodendrocyte Maturation and Delays Learning and Memory Decline in APP/PS1 Mice

**DOI:** 10.3389/fnagi.2020.627362

**Published:** 2021-01-13

**Authors:** Feng-lei Chao, Yi Zhang, Lei Zhang, Lin Jiang, Chun-ni Zhou, Jing Tang, Xin Liang, Jin-hua Fan, Xiao-yun Dou, Yong Tang

**Affiliations:** ^1^Department of Histology and Embryology, Chongqing Medical University, Chongqing, China; ^2^Laboratory of Stem Cells and Tissue Engineering, Chongqing Medical University, Chongqing, China; ^3^Department of Laboratory Medicine, The Second Affiliated Hospital of Chongqing Medical University, Chongqing, China; ^4^Experimental Teaching Management Center, Chongqing Medical University, Chongqing, China; ^5^Department of Physiology, Chongqing Medical University, Chongqing, China; ^6^Academy of Life Sciences, Chongqing Medical University, Chongqing, China

**Keywords:** oligodendrogenesis, oligodendrocytes, hippocampus, APP/PS1 transgenic mice, Alzheimer's disease, fluoxetine

## Abstract

Oligodendrogenesis dysfunction impairs memory consolidation in adult mice, and an oligodendrocyte abnormality is an important change occurring in Alzheimer's disease (AD). While fluoxetine (FLX) is known to delay memory decline in AD models, its effects on hippocampal oligodendrogenesis are unclear. Here, we subjected 8-month-old male amyloid precursor protein (APP)/presenilin 1 (PS1) mice to the FLX intervention for 2 months. Their exploratory behaviors and general activities in a novel environment, spatial learning and memory and working and reference memory were assessed using the open-field test, Morris water maze, and Y maze. Furthermore, changes in hippocampal oligodendrogenesis were investigated using stereology, immunohistochemistry, immunofluorescence staining, and Western blotting techniques. FLX delayed declines in the spatial learning and memory, as well as the working and reference memory of APP/PS1 mice. In addition, APP/PS1 mice exhibited immature hippocampal oligodendrogenesis, and FLX increased the numbers of 2′3′cyclic nucleotide 3′-phosphodiesterase (CNPase)^+^ and newborn CNPase^+^ oligodendrocytes in the hippocampi of APP/PS1 mice. Moreover, FLX increased the density of SRY-related HMG-box 10 protein (SOX10)^+^ cells and reduced the percentage of oligodendrocyte lineage cells displaying the senescence phenotype (CDKN2A/p16INK4a) in the hippocampus of APP/PS1 mice. Moreover, FLX had no effect on the serotonin (5-HT) 1A receptor (5-HT1AR) content or number of 5-HT1AR^+^ oligodendrocytes, but it reduced the content and activity of glycogen synthase kinase 3β (GSK3β) in the hippocampus of APP/PS1 transgenic mice. Taken together, FLX delays the senescence of oligodendrocyte lineage cells and promotes oligodendrocyte maturation in the hippocampus of APP/PS1 mice. FLX may regulate GSK3β through a mechanism other than 5-HT1AR and then inhibit the negative effect of GSK3β on oligodendrocyte maturation in the hippocampus of an AD mouse model.

## Introduction

Alzheimer's disease (AD) is a devastating neurodegenerative disease that is predominantly associated with a progressive cognitive impairment and memory impairment (Alzheimer's Association, 2020). Studies investigating methods to prevent and delay of AD progression are ongoing, and treatment or intervention during the early stage of AD is considered the optimal strategy (Crous-Bou et al., 2017; McDade and Bateman, 2017).

In recent years, the roles of oligodendrocytes in cognitive impairment and neurodegenerative diseases have received considerable attention, particularly in the early stages of AD. Oligodendrocytes metabolically support axon survival and function through mechanisms independent of myelination, and their dysfunction contributes to neurodegeneration (Lee et al., 2012). The generation of myelin-forming oligodendrocytes persists throughout life, and the prevention of oligodendrogenesis was shown to impair memory consolidation in the water maze and contextual fears (Steadman et al., 2020). Myelin and oligodendrocyte abnormalities were determined to be important changes associated with cognitive impairment in the early stages of AD (Bartzokis, 2004; Destrooper and Karran, 2016; Nasrabady et al., 2018; Zhang et al., 2019), and hippocampal pathology is very relevant, particularly in the early stages of the disease (Barnes and Fox, 2014; Ferrarini et al., 2014). Myelin abnormalities and myelinated fibers are decreased in the hippocampus of subjects with AD (Desai et al., 2009; Schmued et al., 2013; Lu et al., 2016; Chao et al., 2018), and these changes occur prior to the appearance of amyloid and tau pathologies (Desai et al., 2009) and hippocampal atrophy (Lu et al., 2016). Wu et al. reported that neuregulin-1 type III, which is required for oligodendrocyte development, was upregulated in the hippocampi of 2-month-old APP/PS1 mice (2017). Hippocampal oligodendrogenesis may be upregulated in AD mice, but researchers have not determine why the total volume of the myelin sheath continues to decrease. However, hippocampal oligodendrogenesis remains to be studied in the early stages of AD, and no study has quantitatively investigated changes in oligodendrocyte in the hippocampus during this stage.

Pathological changes in AD are studied to identify effective targets and strategies for prevention and treatment. Fluoxetine (FLX), a selective serotonin (5-HT) reuptake inhibitor (SSRI) antidepressant, has been shown to improve cognition and memory in studies examining the treatment of psychiatric symptoms in patients with AD (Aboukhatwa et al., 2010; Rozzini et al., 2010; Kim et al., 2013). One clinical study reported that long-term FLX treatment delayed the progression from mild cognitive impairment (MCI) to AD (Mowla et al., 2007), and basic studies showed that FLX also improves the memory and cognition of animals with AD (Ciao et al., 2015; Jin et al., 2017). The main pharmacological effects of FLX are to increase the extracellular 5-HT concentration by inhibiting 5-HT reuptake by synapses, and the effect of 5-HT depends on its binding to its receptor (Dringenberg and Diavolitsis, 2002; Perez-Caballero et al., 2014). According to a previous study, reported that 5-HT1A receptors are expressed in cultured oligodendrocytes at various stages (Azmitia et al., 1996). FLX activates 5-HT1A receptors (5-HT1ARs) in the brains of emotionally impaired mice and schizophrenia model mice by increasing 5-HT concentrations, thereby inhibiting the expression of glycogen synthase kinase 3 (GSK3) (Li et al., 2007; Haroutunian et al., 2014). GSK3β, a subtype of GSK3, is an important negative regulator of oligodendrocyte differentiation and myelination. GSK3β inhibition not only increases the number of oligodendrocytes and oligodendrocyte precursor cells but also promotes the formation of myelin (Azim and Butt, 2011). Lee et al. found that FLX prevented oligodendrocyte cell death after spinal cord injury (Lee et al., 2004). As shown in our previous study, FLX increases the total number of 2′3′-cyclic nucleotide 3′-phosphodiesterase (CNPase)^+^ cells in the hippocampi of rats with chronic unpredictable stress-induced depression (Wang et al., 2020). However, the effects of FLX on oligodendrocytes in the hippocampus during early AD have not been investigated, and in particular, its effects on hippocampal oligodendrogenesis in AD remain unclear.

APP/PS1 transgenic mice, an animal model useful for the study of early AD (Bilkei-Gorzo, 2014), were used here and administered a low-dose FLX treatment for 2 months to address these questions. Their exploratory behaviors and general activities in a novel environment, spatial learning and memory abilities, working and reference memory abilities and changes in hippocampal oligodendrocytes and oligodendrogenesis during early AD, as well as the effects of FLX on these parameters were investigated.

## Materials and Methods

### Animals

The amyloid precursor protein (APP)/presenilin 1 (PS1) transgenic AD mouse model whose genotype was B6C3-Tg (APPswe, PSEN1dE9) 85Dbo/J was used. Eight-month-old male APP/PS1 mice and non-transgenic littermate mice were randomly selected. The transgenic APP/PS1 mice were randomly divided into the normal saline group (APP/PS1 + NaCl, *n* = 14) and the FLX group (APP/PS1 + FLX, *n* = 15). The non-transgenic littermate mice were divided into the normal saline control group (Ctrl + NaCl, *n* = 16) and the FLX control group (Ctrl + FLX, *n* = 15). All mice were provided by the Animal Model Institute of Nanjing University and bred in the Experimental Animal Center at Chongqing Medical University, P. R. China. All animals were housed and maintained at a temperature of 22 ± 1°C and a humidity of 55% in an air-conditioned room (12-h light-dark cycle with the lights on at 8:00 a.m.). All experimental procedures were performed between 0:00 p.m. and 5:00 p.m. Food and water were available *ad libitum*. Animal care and treatment followed the National Institute of Health Guide for the Care and Use of Laboratory Animals (NIH Publication 85–23).

### Intervention Programs

The two FLX groups were subjected to intraperitoneal injections of FLX (Sigma, Germany) at a dose of 10 mg/kg/d for 2 months (Marcussen et al., 2008; Zhou et al., 2019). The two saline groups were subjected to intraperitoneal injections of the same dose of normal saline for 2 months. All mice were intraperitoneally injected with 5-bromo-2′-deoxyuridine (BrdU; Sigma-Aldrich) (50 mg/kg/d) for 5 days during the 5th week of the FLX intervention.

### Open-Field Test

The open-field test (XR-XZ301, Shanghai XinRuan Information Technology Co., Ltd., Shanghai, P. R. China) was performed to evaluate the exploratory behavior and general activity of experimental animals in a novel environment (Prut and Belzung, 2003). After the 2-months intervention, the open-field test was performed in a gray square box. A mouse was placed in a corner of the box at the beginning of the test. The total distance traveled in the open field and the time spent in the inner squares were measured in a 300-s session. The central area activity distance ratio and the central area activity time ratio were calculated.

### Morris Water Maze Test

The Morris water maze test (SLY-WMS, Beijing Sunny Instruments Co., Ltd., Beijing, P. R. China) was applied for six consecutive days to evaluate the hippocampus-dependent spatial learning and memory abilities of each group (Morris, 1984; Chen et al., 2000). The maze consisted of an 8-cm diameter platform and a 90-cm diameter pool filled to a depth of 40 cm with opaque water (22–25°C). Before the formal test began, a visible platform experiment was conducted to observe whether the mice had normal vision and swimming abilities. If not, the mouse was removed. The formal test included the hidden platform test and the probe trial. The hidden platform test was performed every day from the first day to the fifth day, followed by a probe trial test on the sixth day. The swimming speed, escape latency, and swimming distance were recorded during the hidden platform test, and the swimming speed, frequency of target zone crossings, swimming time in the target zone and swimming distance in the target zone were recorded during the probe trial, as reported previously (Chao et al., 2018).

### Y Maze Test

The Y maze test (Xeye Y, Shanghai XinRuan Information Technology Co., Ltd., Shanghai, P. R. China) was used to evaluate the working and reference memory abilities of mice in each group (Dellu et al., 2000). Before the test, the mice were restricted from eating to reduce their body weight by 10–15%. At the beginning of the test, the mice were trained to find the target arm (with a food supply) within 3 days (five training sessions per day for 5 min each), with the target arm being switched every time. After training, the food in the target arm was removed, and the formal test was started (two tests for 5 min each). The numbers of times that the mice correctly and incorrectly identified the target arm were recorded, and correct discrimination ratios were calculated.

### Tissue Processing

Six mice from each group were randomly chosen, anesthetized with 1% pentobarbital sodium (0.4 ml/100 g, Sigma, Germany) and fixed via perfusion with 4% paraformaldehyde (GuangFu Chemical Engineering Institute, P. R. China). Then, the right or left hemisphere was randomly chosen, embedded in optimum cutting temperature compound (OCT, CL-B114, SAKURA, Japan) medium, and coronally sliced at 50-μm equidistant intervals with a cryo-ultramicrotome (CM1950, Leica Microsystems, Germany). According to the stereological sampling principle (Gundersen et al., 1999; Zhao et al., 2013; Zhou et al., 2019), one of every five sections was systematically sampled, with the first section randomly sampled from the first five sections. These sections were used for immunohistochemistry and immunofluorescence staining. The other ten mice in each group were anesthetized with 1% pentobarbital sodium and sacrificed, and hippocampal tissue was isolated and stored at −80°C. These tissues were used for the enzyme-linked immunosorbent assay (ELISA) and Western blot analysis.

### ELISA

The levels of Aβ40 and Aβ42 in hippocampal tissues were measured with an Aβ40 Mouse ELISA Kit (KHB3481, Invitrogen, USA) and an Aβ42 Mouse ELISA Kit (KHB3544, Invitrogen, USA), respectively. First, 50 μl of standards, controls and samples were added to 96-well plates. Second, 50 μl of the Aβ40 or Aβ42 detection antibody solution were added to each well, thoroughly mixed and incubated for 3 h at room temperature, followed by four washes with wash buffer. Third, 100 μl of anti-rabbit IgG horseradish peroxidase (HRP) were added to each well, thoroughly mixed and incubated for 30 min at room temperature, followed by four washes with wash buffer. Fourth, 100 μl of stabilized chromogen were added to each well, thoroughly mixed and incubated for 30 min at room temperature in the dark, and the liquid in the 96-well plates turned blue. Then, 100 μl of stop solution were added and fully mixed, and the liquid changed from blue to yellow. Finally, target protein expression in the plates was quantitatively analyzed at 450 nm.

### Western Blot Analysis

The levels of the target proteins were measured with the following primary antibodies: anti-CNPase (1:1,000, ab6319, Abcam), anti-myelin basic protein (MBP; 1:1,000, ab62631, Abcam), anti-neuron glia 2 (NG2; 1:1,000, sc-33666, SANTA CRUZ), anti-GSK3β (1:1,000, D5C5Z, CST), anti-phosphorylated (Ser9) GSK3β (p-ser9-GSK3β; 1:1,000, D85E12, CST), anti-5-HT1AR (1:1,000, ab85615, Abcam), anti-human gene and protein symbol ACTB/ACTB (β-actin; 1:5,000, ab8226, Abcam), and anti-heat-shock protein 90 (HSP90; 1:2,000, ab203126, Abcam). The corresponding secondary antibodies (goat anti-rabbit IgG, CW0103 and goat anti-rabbit IgG, CW0102, CWBIO, P. R. China) for each protein were applied at a dilution of 1:1,000, followed by development with an ECL kit (AR1111, Boster, P. R. China). The band intensity was quantified using Image Lab software (version 5.2.1).

### Immunohistochemistry

Two groups of sections were randomly selected from each mouse. Immunohistochemistry was performed using Streptavidin/Peroxidase (SP) Link Detection Kits (sp9001 and sp9002, ZSGB-BIO, P. R. China). Briefly, the sections were rinsed with 0.01 M phosphate-buffered saline containing 0.3% Triton X-100 and 0.1% Tween 20 (PBS-T), incubated with 3% hydrogen peroxide for 20 min, antigens were retrieved in citrate buffer (0.01 M, 99°C) for 30 min, and sections incubated in normal goat serum for 2 h at 37°C. Then, the two groups of sections were separately incubated with the primary anti-CNPase (1:500, ab6319, Abcam) and anti-Olig2 (1:500, ab109186, Abcam) antibodies at 4°C for 72 h, incubated with the secondary antibody solutions at 37°C for 2 h, incubated with streptavidin-HRP (S-HRP) at 37°C for 30 min, transferred to a diaminobenzidine solution (DAB, ZLL-9032, ZSGB) for approximately 1 min, washed with deionized water and 0.01 M PBS, and counterstained with hematoxylin (Bude Biological Engineering Co., Ltd., Wuhan, P. R. China). The sections were dehydrated by sequential immersion in a gradient of ethanol solutions, cleared in xylene, air-dried, and sealed with neutral gum.

### Immunofluorescence Staining

A group of sections were randomly selected from the three additional groups of sections from each mouse. The sections were rinsed with PBS-T, successively infiltrated with 2 mol/L HCl for 10 min (only required for the anti-BrdU antibody), subjected to antigen retrieval in citrate buffer (0.01 M, 99°C) for 30 min, and incubated with normal goat serum for 2 h at 37°C. Then, the primary anti-CNPase (ab6319, Abcam), anti-Olig2 (rabbit, ab109186, Abcam), anti-SOX10 (ab155279, Abcam), anti-bromodeoxyuridine (BrdU; ab6326, Abcam), anti-5-HT1AR (ab85615, Abcam), anti-platelet-derived growth factor alpha receptor (PDGFαR; ab96569, Abcam), anti-Aβ (ab11132, Abcam), and anti-CDKN2A/p16INK4a (p16; ab201980, Abcam) antibodies were added at a dilution of 1:500 in PBS, incubated at 4°C for 72 h and then rewarmed at 37°C for 2 h. The appropriate DyLight 405, DyLight 488, and DyLight 549-conjugated secondary antibodies (A23140, A23210, and A23320, respectively, Abbkine, P. R. China) were incubated with the sections at a 1:200 dilution. Finally, the sections were mounted on gelatin-coated slides with antifade solution to reduce fluorescence quenching.

### Quantitative Analyses

**Figure 1:**
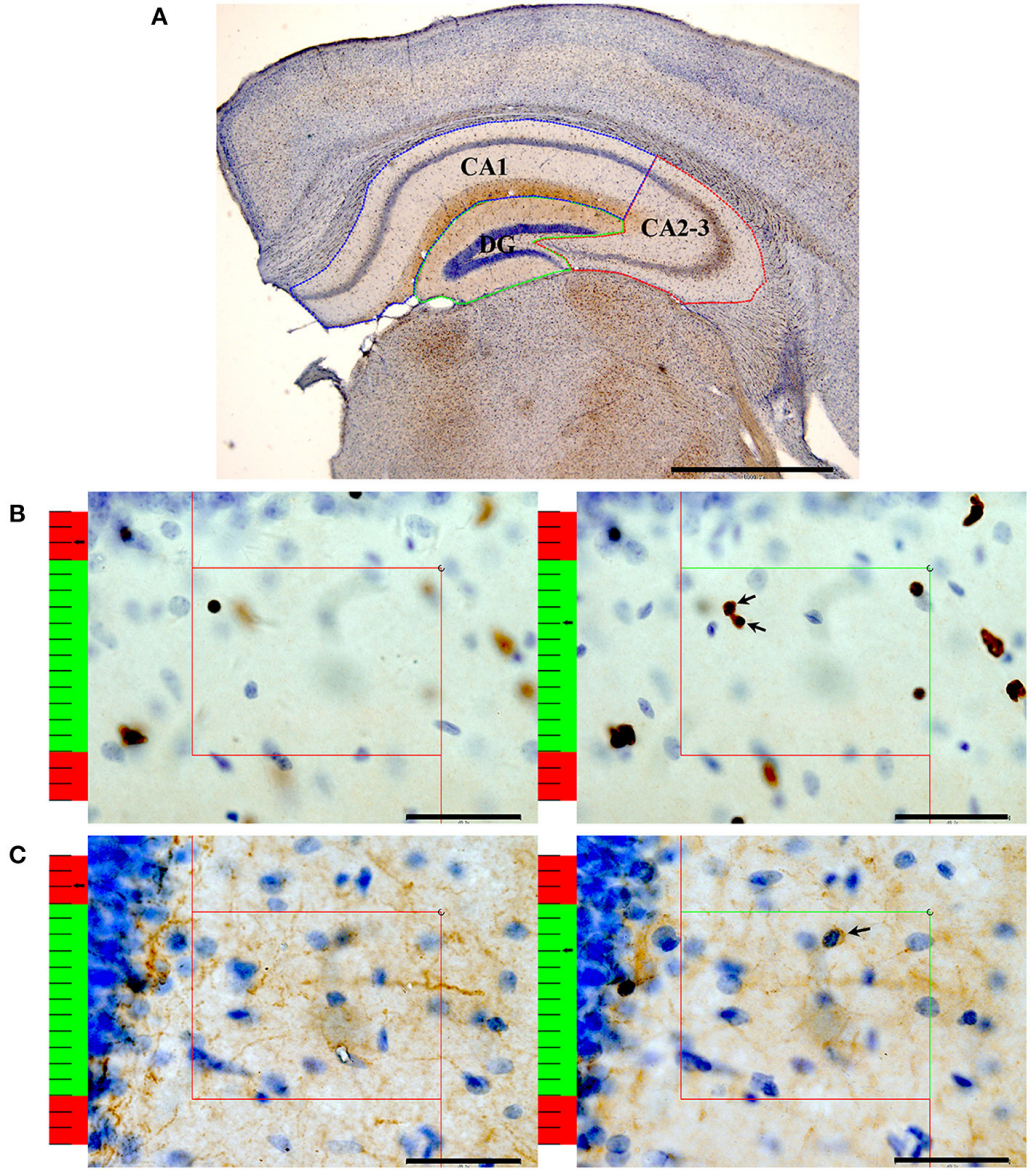
An illustration of the stereological method. **(A)** After performing Olig2 immunohistochemistry, the contours of the CA1 (blue box), CA2-3 (red box) and DG (green box) regions were traced. Bar = 1,000 μm. **(B)** An illustration of the method used to count the number of Olig2^+^ oligodendrocytes with the optical dissector technique. According to the forbidden line rule, the nucleus that is completely in the counting frame or intersects the inclusion lines (green lines) will be counted. If the nucleus intersects the exclusion lines (red lines), it is not counted. On the left: The nucleus is clearly in focus in the guard zone (the red part of the left ruler) and is not counted. On the right: The oligodendrocytes with nuclei clearly in focus in the counting zone (the green part of the left ruler) but not in focus in the guard zone are counted. Arrows show the Olig2^+^ oligodendrocytes that were counted. Bar = 40 μm. **(C)** An illustration of the method used to count the number of CNPase^+^ oligodendrocytes with the optical dissector technique. On the left: The nucleus is clearly in focus in the guard zone and is not counted. On the right: The oligodendrocytes with their nuclei clearly in focus in the counting zone but not in focus in the guard zone are counted. Bar = 40 μm. The arrow shows the CNPase^+^ oligodendrocytes that were counted.

After immunohistochemistry, a computer-assisted morphometry system consisting of a microscope (BX51, Olympus Japan) and stereological analysis software (New CAST, Denmark) was used to count the numbers of oligodendrocyte lineage cells (Olig2^+^ cells) and mature oligodendrocytes (CNPase^+^ cells) in the hippocampal subregions as previously described (Wang et al., 2020; Figure 1). When counting the Olig2^+^ cells, the slice sampling fraction (*ssf)* was 1/5, the area sampling fraction (*asf)* was 6%, and the thickness sampling fraction (*tsf)* was 0.706. When counting the CNPase^+^ cells, the *ssf* was 1/5, the *asf* was 8%, and the *tsf* was 0.681.

After immunofluorescence staining, all quantitative analyses were performed using a laser scanning confocal microscope (Nikon, Japan), and images of the sections were acquired randomly. The numbers of Olig2^+^/CNPase^+^ (mature oligodendrocytes), Olig2^+^/CNPase^−^ (immature oligodendrocytes), and BrdU^+^/Olig2^+^/CNPase^+^ (newborn mature oligodendrocytes) cells in the hippocampal subregions were counted. SOX10 plays key roles in the maturation of oligodendrocytes and myelination (Stolt et al., 2002). Here, the SOX10-positive cells were labeled and the numbers of SOX10^+^ cells in the hippocampal subregions were counted. According to a previous study, β-amyloid induces p16 protein expression in oligodendrocyte progenitor cells and then induces oligodendrocyte progenitor cell senescence (Zhang et al., 2019). Here, senescent oligodendrocytes were labeled, and the ratio of senescent oligodendrocytes (p16^+^/Olig2^+^ cells) in the hippocampal subregions was calculated. Previous studies reported the expression of 5-HT1AR in cultured oligodendrocytes at various stages, and this receptor was shown to play an important regulatory role in oligodendrocyte differentiation and myelination (Azmitia et al., 1996; Li et al., 2007; Haroutunian et al., 2014). Here, 5-HT1AR in oligodendrocyte precursor cells and mature oligodendrocyte was immunofluorescently labeled, and the numbers of oligodendrocyte precursor cells expressing 5-HT1AR (PFDGαR^+^/5-HT1AR^+^ cells) and mature oligodendrocytes expressing 5-HT1AR (CNPase^+^/5-HT1AR^+^ cells) in the hippocampal subregions were counted.

### Statistics

All statistical analyses were performed using SPSS (ver. 19.0, SPSS Inc., Chicago, USA). The Shapiro-Wilk test was used to evaluate whether the group means of data were normally distributed. Repeated-measures analysis of variance (ANOVA) was used to analyze body weight, swimming speed, escape latency and swimming distance. For the remaining data, one-way ANOVA followed by the LSD *post hoc* test were used for the analysis. A *p* < 0.05 was considered to indicate a significant difference. The observed coefficient of variation (OCV) values and observed coefficient of error (OCE) values were calculated using the method reported by Gundersen et al. (1988, 1999).

## Results

### The FLX Intervention Did Not Negatively Affect the Body Weight of Mice

Before the intervention, the body weight of mice in the Ctrl + NaCl group was 37.71 ± 3.50 g, the body weight of mice in the Ctrl + FLX group was 38.75 ± 4.08 g, the body weight of mice in the APP/PS1 + NaCl group was 37.76 ± 5.35 g, and the body weight of mice in the APP/PS1 + FLX group was 38.39 ± 4.15 g. After the intervention, the body weight of mice in the Ctrl + NaCl group was 36.64 ± 3.26 g, the body weight of mice in the Ctrl + FLX group was 36.49 ± 4.11 g, the body weight of mice in the APP/PS1 + NaCl group was 37.39 ± 5.29 g, and the body weight of mice in the APP/PS1 + FLX group was 38.07 ± 4.84 g. During the intervention, a significant difference in the body weight of mice was not observed among the Ctrl + NaCl, Ctrl + FLX, APP/PS1 + NaCl, and APP/PS1 + FLX groups (*F* = 0.112, *p* = 0.953).

### The FLX Intervention Did Not Negatively Affect the Exploratory Behavior or General Activity of Mice in a Novel Environment

After the intervention, no significant differences in the central area activity distance ratio or in the central area activity time ratio were observed among the Ctrl + NaCl, Ctrl + FLX, APP/PS1 + NaCl, and APP/PS1 + FLX groups (*F* = 0.287, *p* = 0.835 and *F* = 0.453, *p* = 0.716, Table 1).

**Table 1:** The results (mean ± SEM) of behavioral tests.

		**Ctrl + NaCl *n =* 16**	**Ctrl + FLX *n =* 15**	**APP/PS1 + NaCl *n =* 14**	**APP/PS1 + FLX *n =* 15**
Open-field	Central area activity distance ratio (%)	13.55 ± 1.48	12.56 ± 1.25	13.97 ± 0.99	12.67 ± 1.07
test	Central area activity time ratio (%)	11.78 ± 1.93	9.53 ± 1.32	11.11 ± 1.20	11.65 ± 1.66
	Swimming speed in day 1 (cm/s)	15.35 ± 1.38	14.14 ± 1.14	13.62 ± 0.87	12.67 ± 0.70
	Swimming speed in day 2 (cm/s)	14.78 ± 1.03	13.62 ± 1.19	14.24 ± 0.84	12.8 ± 0.88
Morris	Swimming speed in day 3 (cm/s)	13.90 ± 0.83	13.37 ± 1.20	14.40 ± 0.72	13.72 ± 0.95
water maze	Swimming speed in day 4 (cm/s)	12.58 ± 0.92	13.07 ± 0.84	13.61 ± 0.90	13.42 ± 0.83
test	Swimming speed in day 5 (cm/s)	12.05 ± 0.76	12.55 ± 1.08	13.46 ± 0.88	13.20 ± 0.66
	Swimming speed in day 6 (cm/s)	14.25 ± 1.18	14.15 ± 1.01	14.79 ± 0.92	14.46 ± 0.95
	Swimming time in target zone in day 6 (s)	27.72 ± 3.50	33.59 ± 3.95	34.52 ± 3.17	28.92 ± 3.16
	Swimming distance in target zone in day 6 (m)	2.23 ± 0.32	2.96 ± 0.22	2.83 ± 0.34	2.61 ± 0.27

### FLX Delayed Deficiencies in the Spatial Learning and Memory Abilities of Mice in the Early Stages of AD

During the Morris water maze test, no significant differences in swimming speed were observed among the four groups (*F* = 0.144, *p* = 0.933; Table 1). The FLX intervention did not exert a negative effect on the swimming ability of mice.

**Figure 2:**
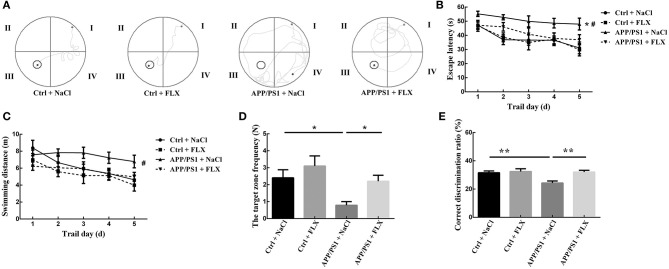
The effects of FLX on the spatial learning and memory abilities, as well as working and reference memory abilities of APP/PS1 mice. **(A)** Tracked locations of the mice in the hidden platform test. The Roman numerals (I, II, III, and IV) represent the first, second, third, and fourth quadrants of the Morris water maze, respectively. **(B)** Escape latencies in the Morris water maze positioning navigation test. Each point represents the average of the four escape latencies (x ± SEM). *p < 0.05 for the comparison of the escape latency of the Ctrl + NaCl group with the APP/PS1 + NaCl group in the Morris water maze positioning navigation test. #*p* < 0.05 for the comparison of the escape latency of the APP/PS1 + NaCl group with the APP/PS1 + FLX group in the Morris water maze positioning navigation test. **(C)** Swimming distance in the Morris water maze positioning navigation test. Each point represents the average of the four escape latencies (x ± SEM). #*p* < 0.05 for the comparison of the escape latency of the APP/PS1 + NaCl group with the APP/PS1 + FLX group in the Morris water maze positioning navigation test. **(D)** Target zone frequency in the probe trial tests (x ± SEM). *p < 0.05. **(E)** Correct discrimination ratio in the Y maze (mean ± SEM). **p < 0.01.

The location traces of the mice in the four groups during the hidden platform test are presented in Figure 2A. Significant differences in the escape latency times and swimming distances were observed among the four groups (*F* = 5.029, *p* = 0.004 and *F* = 2.79, *p* = 0.049; Figures 2B,C). First, the escape latency of the APP/PS1 + NaCl group was significantly longer than the Ctrl + NaCl group (*p* = 0.001; Figure 2B). Second, no significant differences in the escape latency and swimming distance were observed between the Ctrl + NaCl group and Ctrl + FLX group (*p* = 0.954 and *p* = 0.272; Figures 2B,C). Third, the escape latency and swimming distance of the APP/PS1 + FLX group were significantly shorter than the APP/PS1 + NaCl group (*p* = 0.024 and *p* = 0.027; Figures 2B,C).

During the probe trial, significant differences in the swimming time and swimming distance in the target zone were not observed among the four groups of mice (*F* = 0.94, *p* = 0.428 and *F* = 1.146, *p* = 0.338; Table 1); however, a significant difference in the target zone frequency was observed among the four groups (*F* = 4.604, *p* = 0.006; Figure 2D). First, the target zone frequency of mice in the APP/PS1 + NaCl group was significantly lower than mice in the Ctrl + NaCl group (*p* = 0.013; Figure 2D). Second, no significant difference in the target zone frequency was identified between the Ctrl + NaCl group and Ctrl + FLX group (*p* = 0.266; Figure 2D). Third, the target zone frequency of the APP/PS1 + NaCl group was significantly lower than the APP/PS1 + FLX group (*p* = 0.031; Figure 2D).

### FLX Delayed Deficiencies in the Working and Reference Memory Abilities of Mice in the Early Stages of AD

Significant differences in the correct discrimination ratios were observed among the four groups (*F* = 6.475, *p* = 0.001; Figure 2E). First, the correct discrimination ratio of mice in the APP/PS1 + NaCl group was significantly lower than mice in both the Ctrl + NaCl group and the APP/PS1 + FLX group (*p* = 0.001 and *p* = 0.001; Figure 2E). Second, a significant difference in the correct discrimination ratios was not observed between the Ctrl + NaCl and Ctrl + FLX groups (*p* = 0.678; Figure 2E).

### FLX Affected the Levels of Myelin-Related Proteins in the Hippocampi of AD Mice

**Figure 3:**
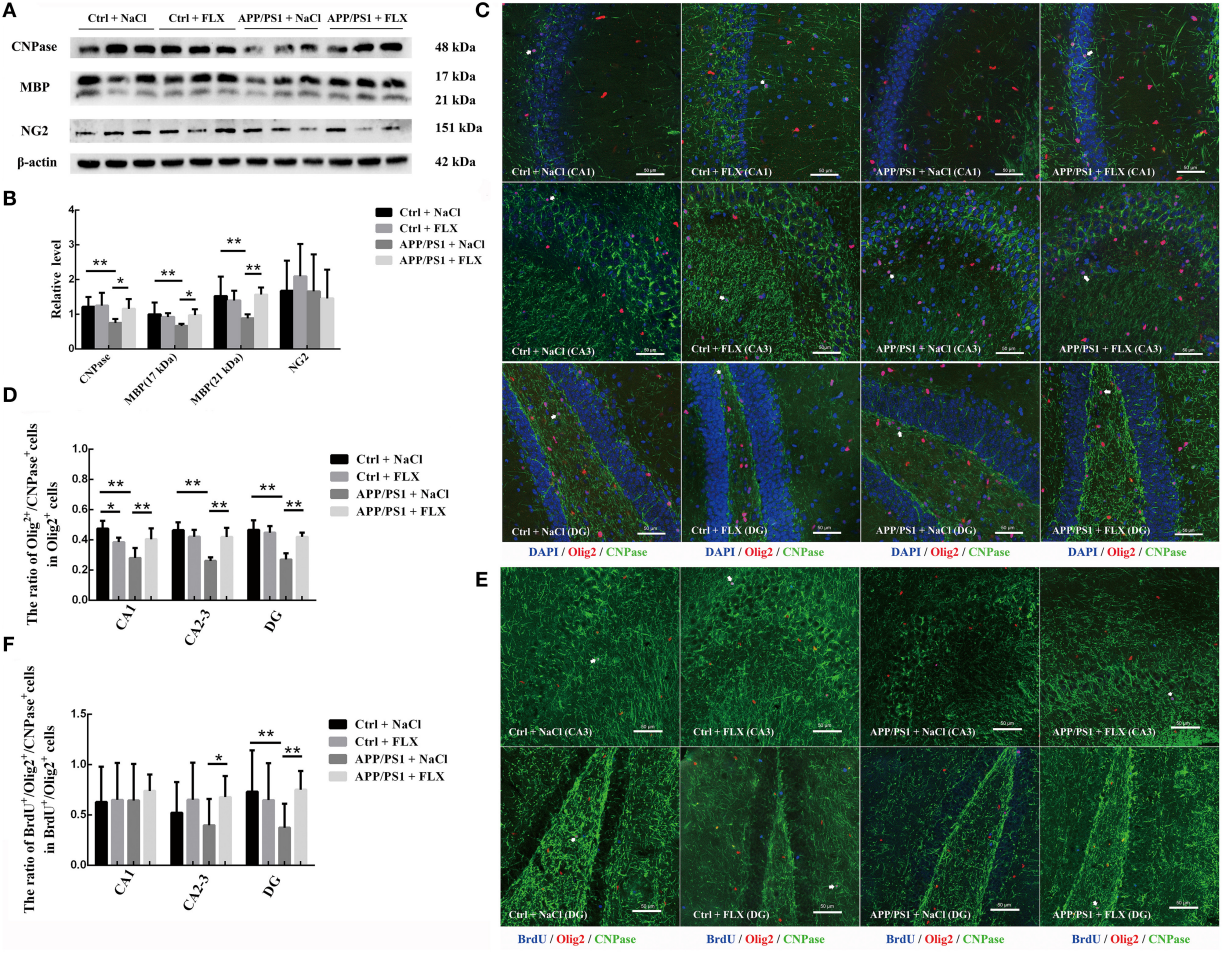
The effects of FLX on the oligodendrocytes in the hippocampus of APP/PS1 mice. **(A)** Western blots showing the levels of CNPase, MBP and NG2 in the mouse hippocampus. **(B)** The relative levels of the CNPase, MBP and NG2 proteins in the hippocampus (x ± SD). **(C)** Immunofluorescence staining of mature oligodendrocytes (Olig2^+^/CNPase^+^ cells) in the hippocampus. Arrows (→) indicate Olig2^+^/CNPase^+^ cells. Bar = 50 μm. **(D)** The ratio of mature oligodendrocytes to oligodendrocyte lineage cells in the hippocampus (x ± SD). **(E)** Immunofluorescence staining of immature newborn mature oligodendrocytes (BrdU^+^/Olig2^+^/CNPase^+^ cells) in the hippocampus. Arrows (→) indicate BrdU^+^/Olig2^+^/CNPase^+^ cells. Bar = 50 μm. **(F)** The ratio of newborn mature oligodendrocytes to newborn oligodendrocyte lineage cells in the hippocampus (x ± SD). *p < 0.05. **p < 0.01.

We semiquantitatively analyzed the levels of the CNPase, MBP, and NG2 proteins in the hippocampi of mice from the four groups (Figure 3A). We observed significant differences in the hippocampal levels of CNPase and MBP (17 and 21 kDa) among the four groups (*F* = 4.244, *p* = 0.018; *F* = 3.575, *p* = 0.032 and *F* = 5.323, *p* = 0.007, respectively). Compared with the non-transgenic mice, the APP/PS1 transgenic mice (10 months old) displayed hippocampal protein levels of CNPase and MBP (17 kDa and 21 kDa) (*p* = 0.008, *p* = 0.008 and *p* = 0.003, respectively; Figure 3B), and FLX increased the hippocampal protein levels of CNPase and MBP (17 kDa and 21 kDa) in the APP/PS1 transgenic mice (*p* = 0.019, *p* = 0.015, and *p* = 0.002, respectively; Figure 3B). However, a significant difference in the hippocampal level of NG was not observed in mice among the four groups (*F* = 0.497, *p* = 0.688; Figure 3B).

### FLX Rescued the Decreased Number of Mature Oligodendrocytes and Reversed the Abnormal Increase in the Number of Oligodendrocyte Lineage Cells in the Hippocampi of AD Mice

Using unbiased three-dimensional stereological methods, we counted the total numbers of Olig2^+^ cells (oligodendrocyte lineage cells) and CNPase^+^ cells (mature oligodendrocytes) in the hippocampus (sampling information is shown in Table 2; results are shown in Table 3). In the current study, the square of the observed coefficient of error variance (OCE^2^) of the individual estimate was less than half of the observed interindividual variance (OCV^2^), indicating that the sampling was optimal. Here, significant differences in the total numbers of Olig2^+^ cells in the CA1 field, CA2-3 fields and DG were observed among the four groups (*F* = 5.239, *p* = 0.008; *F* = 6.215, *p* = 0.004 and *F* = 14.759, *p* < 0.001, respectively; Table 3) and in the total numbers of CNPase^+^ cells in the CA1 field, CA2-3 fields and DG among the four groups (*F* = 7.956, *p* = 0.001; *F* = 5.368, *p* = 0.007 and *F* = 10.442, *p* < 0.001, respectively; Table 3). First, compared with the non-transgenic mice, the APP/PS1 transgenic mice (10 months old) exhibited a significant increase in the numbers of Olig2^+^ cells in the CA1 field, CA2-3 fields and DG (*p* = 0.037, *p* = 0.002, and *p* < 0.001, respectively; Table 3), and the FLX treatment significantly reduced the total number of Olig2^+^ cells in the CA2-3 fields and DG of the APP/PS1 transgenic mice (*p* = 0.021 and *p* = 0.003; Table 3). Second, compared with the non-transgenic mice, the APP/PS1 transgenic mice (10 months old) displayed a significant decrease in the numbers of CNPase^+^ cells in the CA1 field, CA2-3 fields and DG (*p* = 0.003, *p* = 0.012 and *p* < 0.001, respectively; Table 3), and FLX treatment significantly increased the total numbers of CNPase^+^ cells in the CA1 field, CA2-3 fields and DG of the APP/PS1 transgenic mice (*p* = 0.001, *p* = 0.005, and *p* < 0.001, respectively; Table 3).

**Table 2:** The sampling information of quantitative analyses.

		**Stereological quantification**						**Laser confocal scanning microscopy quantification**					
		**(*****n=*** **6/group)**						**(*****n*** **= 5/group)**					
		**Olig2**			**CNPase**			**Olig2/CNPase**			**BrdU/Olig2/CNPase**		
		**CA1**	**CA2-3**	**DG**	**CA1**	**CA2-3**	**DG**	**CA1**	**CA2-3**	**DG**	**CA1**	**CA2-3**	**DG**
Ctrl	N (animals)	6	6	6	6	6	6	5	5	5	4	4	4
+ NaCl	N (sections)	68	63	72	57	56	63	22	25	28	24	20	23
	N (views)	924	619	657	890	675	652	116	129	136	48	52	53
Ctrl	N (animals)	6	6	6	6	6	6	5	5	5	4	4	4
+ FLX	N (sections)	72	62	75	65	59	75	24	25	27	15	15	15
	N (views)	1,019	600	698	980	560	688	132	131	149	47	58	58
APP/PS1	N (animals)	6	6	6	6	6	6	5	5	5	4	4	4
+ NaCl	N (sections)	65	62	74	70	65	75	23	19	24	24	24	24
	N (views)	904	562	546	1094	657	706	113	101	131	40	48	71
APP/PS1	N (animals)	6	6	6	6	6	6	5	5	5	4	4	4
+ FLX	N (sections)	67	54	66	51	49	59	26	22	24	24	24	24
	N (views)	919	599	673	1067	670	665	132	117	118	52	46	53

*N (animals) is the total number of animals that were analyzed from each group. N (sections) is the total number of sections that were analyzed from each group. N (views) is the total number of views that were analyzed from each group*.

**Table 3:** Stereological results of the oligodendrocytes in the hippocampus.

		**Total number of the positive oligodendrocytes in the hippocampus (*****n=*** **5/group)**					
		**Olig2 positive oligodendrocytes**			**CNPase positive oligodendrocytes**		
		**CA1**	**CA2-3**	**DG**	**CA1**	**CA2-3**	**DG**
Ctrl	Mean (× 10^4^)	10.22	10.47	7.82	4.26	3.45	3.84
+ NaCl	SD	1.59	0.78	0.57	0.83	0.95	0.76
	OCV (%)	15.55	7.49	7.32	19.52	27.63	19.92
	OCE (%)	4.56	4.57	5.21	6.66	7.49	7.02
Ctrl + FLX	Mean (× 10^4^)	8.72	10.32	7.41	4.68	3.84	3.93
	SD	1.95	1.52	0.27	0.91	0.69	0.60
	OCV (%)	22.36	14.73	3.70	19.47	18.01	15.36
	OCE (%)	5.21	5.03	5.43	6.30	6.94	6.83
APP/PS1	Mean (× 10^4^)	12.31^*^	13.32^**^	9.88^**^	2.65^**^	2.21^*^	2.27^**^
+ NaCl	SD	1.28	1.24	0.55	0.28	0.49	0.40
	OCV (%)	10.45	9.34	5.65	10.67	22.45	17.98
	OCE (%)	4.43	4.36	4.99	8.40	9.37	9.13
APP/PS1	mean (× 10^4^)	11.08	11.36^#^	8.51^##^	4.50^##^	3.61^##^	3.82^##^
+ FLX	SD	1.55	1.69	1.09	0.90	0.85	0.58
	OCV (%)	14.01	14.94	12.86	20.09	23.74	15.19
	OCE (%)	4.18	4.19	5.43	6.56	7.35	7.06

*Mean is the mean value of the total number of positive oligodendrocytes in the hippocampus of each group. SD is the standard deviation of the mean, OCV is the observed coefficient of variation in each group. OCE is the observed coefficient of error in each group*.

* *p < 0.05 vs. the Ctrl+NaCl group*.

** *p < 0.01 vs. the Ctrl+NaCl group*.

# *p < 0.05 vs. the APP/PS1+NaCl group*.

## *p < 0.01 vs. the APP/PS1+NaCl group*.

Using double-labeling immunofluorescence, we counted the Olig2^+^ (oligodendrocyte lineage cells), Olig2^+^/CNPase^+^ (mature oligodendrocyte), and Olig2^+^/CNPase^−^ (immature oligodendrocyte) cells in the hippocampus (CA1, CA2-3, and DG) of the four groups (sampling information is shown in Table 2; results are shown in Table 4). Significant differences in the densities of Olig2^+^/CNPase^+^ cells were observed in the CA1 field, CA2-3 fields, and DG among the four groups (*F* = 12.157, *p* < 0.001; *F* = 5.599, *p* = 0.008 and *F* = 3.774, *p* = 0.032, respectively; Table 4). First, the APP/PS1 transgenic mice (10 months old) exhibited a significant decrease in the numbers of mature oligodendrocytes within the CA1 field, CA2-3 fields and DG compared to non-transgenic littermates (*p* = 0.001, *p* < 0.001, and *p* < 0.001, respective; Table 4 and Figure 3C). Second, in normal mice, the FLX treatment significantly decreased the number of mature oligodendrocytes in the CA1 field (*p* = 0.015; Table 4 and Figure 3C); however, in APP/PS1 transgenic mice, the FLX treatment significantly rescued the decrease in the numbers of mature oligodendrocytes in the CA1 field, CA2-3 fields and DG (*p* = 0.007, *p* = 0.011, and *p* = 0.014, respective; Table 4 and Figure 3C). Moreover, we identified significant differences in the ratios of mature oligodendrocyte to oligodendrocyte lineage cells in the CA1 field, CA2-3 fields and DG among the four groups (*F* = 9.808, *p* = 0.001; *F* = 17.789, *p* < 0.001 and *F* = 19.451, *p* < 0.001, respectively; Figure 3D). First, the ratios of mature oligodendrocyte to oligodendrocyte lineage cells in the CA1 field, CA2-3 fields and DG of the APP/PS1 + NaCl mice were significantly reduced by 19.51, 20.28, and 19.66%, respectively, compared with the Ctrl + NaCl mice (*p* < 0.001, *p* < 0.001, and *p* < 0.001, respectively; Figure 3D). Second, the FLX treatment significantly decreased the ratio of mature oligodendrocyte to oligodendrocyte lineage cells within the CA1 field of the normal mice by 8.99% (*p* = 0.025; Figure 3D), but increased the ratios of mature oligodendrocyte to oligodendrocyte lineage cells within the CA1 field, CA2-3 fields and DG of the AD mice by 12.40, 15.83, and 14.93%, respectively (*p* = 0.004, *p* < 0.001, and *p* < 0.001, respectively; Figure 3D).

**Table 4:** The results of the oligodendrocytes in the hippocampus using laser confocal scanning microscopy quantification.

		**Density of the positive oligodendrocytes in the hippocampus (*****n=*** **5/group)**								
		**Olig2**^**+**^ **cells**			**Olig2**^**+**^**/CNPase**^**+**^ **cells**			**Olig2**^**+**^**/CNPase**^**−**^ **cells**		
		**CA1**	**CA2-3**	**DG**	**CA1**	**CA2-3**	**DG**	**CA1**	**CA2-3**	**DG**
Ctrl + NaCl	Mean (per mm^2^)	151.82	268.68	198.56	71.61	124.28	91.25	80.21	144.41	107.31
	SD	13.11	45.61	45.11	4.24	24.08	17.80	13.85	30.55	31.06
Ctrl + FLX	Mean (per mm^2^)	160.42	264.28	199.57	61.35	110.83	89.92	99.07	153.44	109.94
	SD	20.22	21.83	17.10	5.03	13.36	15.75	16.28	19.44	4.74
APP/PS1 + NaCl	Mean (per mm^2^)	177.53^*^	316.43^*^	257.37^*^	48.79^*^	82.10^*^	68.73^*^	128.73^*^	234.33^*^	188.64^*^
	SD	21.21	27.75	29.38	8.10	5.06	6.22	25.46	26.72	30.05
APP/PS1 + FLX	Mean (per mm^2^)	152.30^**#**^	270.88^**#**^	217.32	60.58^**#**^	113.20^**#**^	90.66^**#**^	91.72^**#**^	157.68^**#**^	126.66^**#**^
	SD	18.15	31.69	23.72	5.86	19.28	5.68	20.62	27.13	19.30

*Mean is the average density of the positive cells in the hippocampus of each group. SD is the standard deviation of the mean*.

* *p < 0.05 vs. the Ctrl+NaCl group*.

# *p < 0.05 vs. the APP/PS1+NaCl group*.

### FLX Increased the Number of Newborn Mature Oligodendrocytes in the Hippocampi of AD Mice

We counted the BrdU^+^/Olig2^+^ (newborn oligodendrocyte lineage cells) cells and BrdU^+^/Olig2^+^/CNPase^+^ (newborn mature oligodendrocytes) cells in the hippocampus (CA1, CA2-3, and DG) of the four groups of mice to evaluate the regeneration of oligodendrocytes using three-label immunofluorescence staining (sampling information is shown in Table 2; results are shown in Table 5). Significant differences in the densities of newborn mature oligodendrocytes were observed in the CA2-3 fields and DG among the four groups (*F* = 5.859, *p* = 0.011 and *F* = 15.712, *p* < 0.001; Table 5 and Figure 3E) and in the ratios of newborn mature oligodendrocytes to newborn oligodendrocyte lineage cells in the CA2-3 fields and DG among the four groups (*F* = 4.375, *p* = 0.027 and *F* = 23.471, *p* < 0.001; Table 5 and Figure 3E). First, the density of BrdU^+^/Olig2^+^/CNPase^+^ cells in the DG of the APP/PS1 transgenic mice was significantly lower than in the non-transgenic littermates (*p* = 0.015; Table 5 and Figure 3E). In addition, the ratio of newborn mature oligodendrocytes to newborn oligodendrocyte lineage cells in the DG of the APP/PS1 transgenic mice was significantly reduced by 44.63% compared with the non-transgenic littermates (*p* < 0.001; Figure 3F). Second, the FLX treatment significantly increased the density of BrdU^+^/Olig2^+^/CNPase^+^ cells in the CA2-3 fields (*p* = 0.04) of the normal mice (*p* = 0.021; Table 5 and Figure 3E) and produced 24.69 and 36.49% increases in the ratios of newborn mature oligodendrocytes to newborn oligodendrocyte lineage cells within the CA2-3 field and DG, respectively, in the APP/PS1 transgenic mice (*p* < 0.001; Figure 3F).

**Table 5:** The density of the newborn oligodendrocytes in the hippocampus using laser confocal scanning microscopy semiquantification.

		**Density of the newborn oligodendrocytes in the hippocampus (***n =*** 5/group)**					
		**BrdU**^**+**^**/Olig2**^**+**^ **cells**			**BrdU**^**+**^**/Olig2**^**+**^**/CNPase**^**+**^ **cells**		
		**CA1**	**CA2-3**	**DG**	**CA1**	**CA2-3**	**DG**
Ctrl	Mean (per mm^2^)	15.24	22.67	19.62	11.94	14.67	17.90
+ NaCl	SD	1.16	2.41	0.58	0.81	1.56	0.47
Ctrl	Mean (per mm^2^)	15.05	23.19	19.62	12.26	18.85^∧^	15.87
+ FLX	SD	1.16	1.52	0.71	1.81	1.43	1.45
APP/PS1	Mean (per mm^2^)	14.49	23.06	21.40	11.64	11.27	9.73^*^
+ NaCl	SD	0.65	1.97	2.72	0.76	2.95	1.66
APP/PS1	Mean (per mm^2^)	16.04	19.87	15.95^#^	13.01	14.40	13.31^#^
+ FLX	SD	1.82	4.58	2.83	1.52	1.82	2.75

*Mean is the average density of the newborn oligodendrocytes in the hippocampus of each group, SD is the standard deviation of the mean*.

* *p < 0.05 for the comparison of the densities of the positive oligodendrocytes between the Ctrl + NaCl group and the APP/PS1 + NaCl group*.

# *p < 0.05 for the comparison of the densities of the positive oligodendrocytes between the APP/PS1 + NaCl group and the APP/PS1 + FLX group*.

∧ *p < 0.05 for the comparison of the densities of the positive oligodendrocytes between the Ctrl + NaCl group and the Ctrl + NaCl group*.

### FLX Increased the Number of SOX10^+^ Oligodendrocytes in the Hippocampi of AD Mice

**Figure 4:**
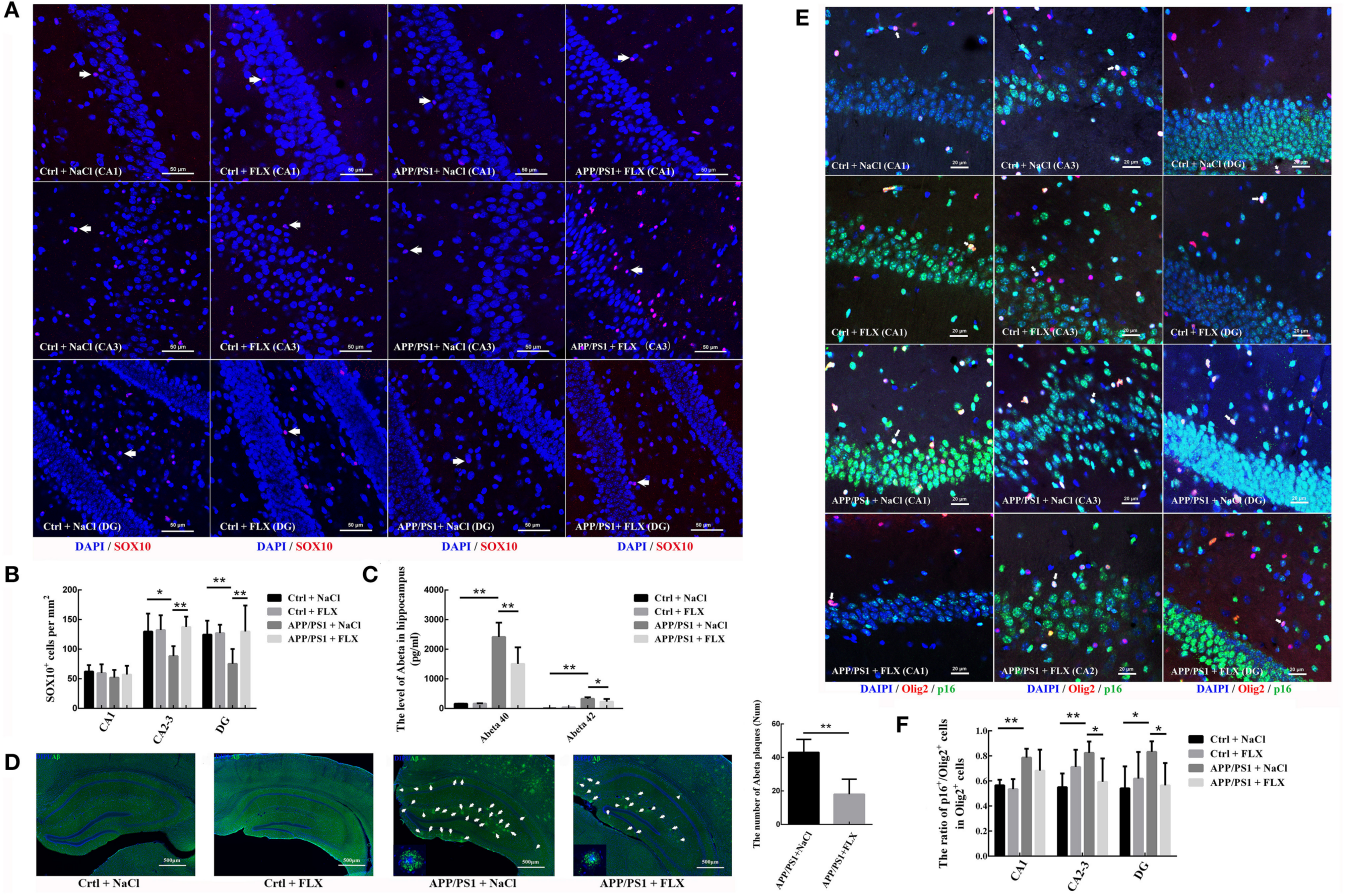
The effects of FLX on the late differentiation and senescence of oligodendrocytes in the hippocampus of APP/PS1 mice. **(A)** Immunofluorescence staining of SOX10^+^ cells in the hippocampus. Arrows (→) indicate the SOX10^+^ cells. Bar = 50 μm. **(B)** Density of SOX10^+^ cells in the hippocampus (x ± SD). **(C)** ELISA results for soluble human Aβ40 and Aβ42 levels (x ± SD). **(D)** Immunofluorescence-based morphology of Aβ in the hippocampus and the number of Aβ plaques with a diameter larger than 20 μm. Aβ plaques were deposited in the hippocampi of APP/PS1 mice, and FLX ameliorated Aβ plaque deposition. Arrows (→) indicate Aβ plaques in the hippocampus. High-magnification images of Aβ staining in 10-month-old APP/PS1 + NaCl and APP/PS1 + FLX mice are shown in each left corner. Bar = 500 μm. **(E)** Immunofluorescence staining of p16^+^/Olig2^+^ cells in the hippocampus. Arrows (→) indicate p16^+^/Olig2^+^ cells. Bar = 20 μm. **(F)** The ratio of p16^+^ oligodendrocytes to oligodendrocyte lineage cells in the hippocampus (x ± SD). *p < 0.05. **p < 0.01.

SOX10 plays key roles in the maturation of oligodendrocytes and myelination. In this study, using double-labeling immunofluorescence, we sampled an average of 76 views from each group to observe the level of the SOX10 protein in the hippocampus (CA1, CA2-3, and DG) of the four groups (Figure 4A). Significant differences in the densities of SOX10^+^ cells were observed in the CA2-3 fields and DG among the four groups (*F* = 4.829, *p* = 0.014 and *F* = 4.088, *p* = 0.025; Figure 4B). The APP/PS1 transgenic mice (10 months old) exhibited significantly lower levels of SOX10 in the CA2-3 fields and DG (88.19 ± 16.91 per mm^2^ in CA2-3 fields and 72.23 ± 24.95 per mm^2^ in DG) than non-transgenic littermates (129.70 ± 30.41 per mm^2^ in CA2-3 fields and 124.51 ± 23.56 per mm^2^ in DG; *p* = 0.012 and *p* = 0.016; Figure 4B), and the FLX treatment significantly increased the level of SOX10 in the CA2-3 fields and DG of the APP/PS1 transgenic mice (137.48 ± 17.40 per mm^2^ in CA2-3 fields and 129.70 ± 43.98 per mm^2^ in DG; *p* = 0.004 and *p* = 0.009; Figure 4B).

### FLX Reduced the Levels of Soluble Aβ40 and Aβ42, the Number of β-Amyloid Plaques and the Percentage of Oligodendrocytes Expressing p16 in the Hippocampi of AD Mice

In this study, we analyzed β-amyloid levels in the hippocampus. Significant differences in the levels of beta amyloid peptide 40 (Aβ40) and Aβ42 in the hippocampus were observed among the four groups (*F* = 39.666, *p* < 0.001 and *F* = 34.777, *p* < 0.001; Figure 4C). The APP/PS1 transgenic mice (10 months old) presented significantly higher levels of Aβ40 and Aβ42 in the hippocampus than non-transgenic littermates (*p* < 0.001 and *p* < 0.001; Figure 4C), and the FLX treatment significantly reduced the levels of Aβ40 and Aβ42 in the hippocampi of the APP/PS1 transgenic mice (*p* = 0.006 and *p* = 0.018; Figure 4C). In addition, no amyloid plaques were detected in the hippocampi of the non-transgenic littermate mice, whereas many large amyloid plaques were observed in the hippocampi of the APP/PS1 transgenic mice, and FLX reduced amyloid plaques (larger than 20 microns in diameter) in the hippocampi of the APP/PS1 transgenic mice (*p* = 0.002, Figure 4D).

Using double-labeling immunofluorescence staining, we sampled an average of 121 views from each group to observe the level of the p16 protein in hippocampal oligodendrocytes (Figure 4E). Here, significant differences in the ratios of oligodendrocytes expressing p16 in the CA1 field, CA2-3 fields and DG were observed among the four groups (*F* = 5.687, *p* = 0.01; *F* = 4.632, *p* = 0.016 and *F* = 3.144, *p* = 0.049, respectively; Figure 4F). The ratios of oligodendrocytes expressing p16 in the CA1 field, CA2-3 fields and DG were significantly higher in APP/PS1 transgenic mice (78.63 ± 7.06% in the CA1 field, 82.38 ± 9.11% in the CA2-3 fields and 83.18 ± 8.41% in the DG) than in non-transgenic littermate controls (56.57 ± 4.35% in the CA1 field, 55.01 ± 10.94% in the CA2-3 fields and 54.11 ± 17.54% in the DG; *p* = 0.004, *p* = 0.003, and *p* = 0.01, respectively; Figure 4F). The FLX treatment significantly decreased the ratios of oligodendrocytes expressing p16 in the CA2-3 fields and DG of AD mice (59.30 ± 18.67% in the CA2-3 fields and 56.45 ± 17.80% in the DG; *p* = 0.018 and *p* = 0.024, respectively; Figure 4F).

### FLX Reduced the Protein Content and Activity of GSK3β but Had No Effect on 5HT1AR Expression in the Hippocampi of Mice With AD

**Figure 5:**
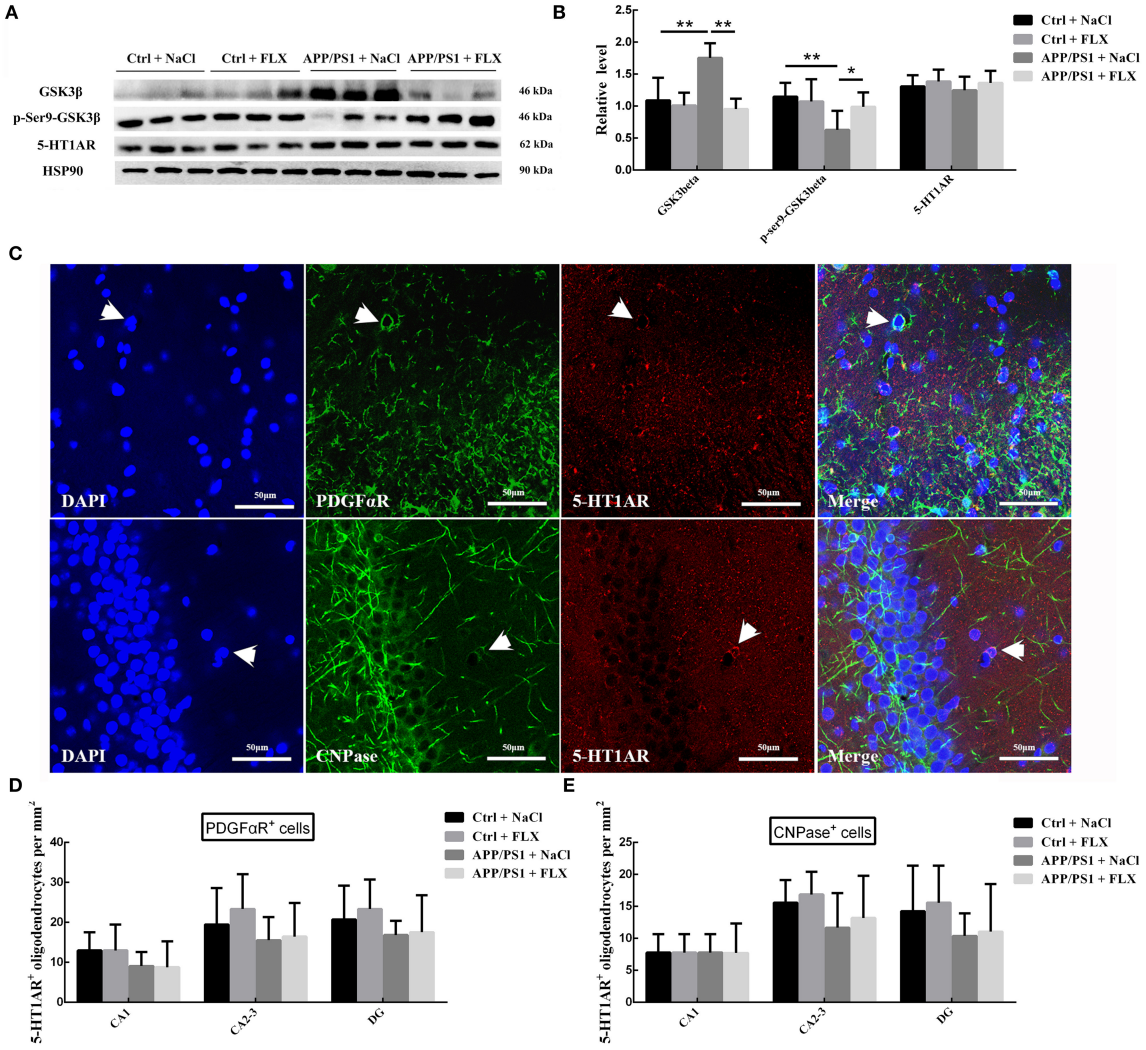
The effects of FLX on GSK3β and 5-HT1AR levels in the hippocampus of APP/PS1 mice. **(A)** Western blots showing the levels of GSK3β, p-S9-GSK3β and 5-HT1AR in the hippocampus. **(B)** The relative levels of the GSK3β, p-S9-GSK3β and 5-HT1AR proteins in the hippocampus (x ± SD). *p < 0.05. **p < 0.01. **(C)** Immunofluorescence staining for 5-HT1AR in oligodendrocyte precursor cells (5-HT1AR^+^/PFDGαR^+^ cells, indicated with arrows) and mature oligodendrocytes (5-HT1AR^+^/CNPase^+^ cells, indicated with arrows) in the hippocampus. Bar = 50 μm. **(D)** The densities (x ± SD) of oligodendrocyte precursor cells expressing 5-HT1AR (5-HT1AR^+^/PFDGαR^+^ cells) in the hippocampus. **(E)** The densities (x ± SD) of mature oligodendrocytes expressing 5-HT1AR (5-HT1AR^+^/CNPase^+^ cells) in the hippocampus.

We semiquantitatively analyzed the hippocampal levels of the GSK3β and p-Ser9-GSK3β proteins in mice from the four groups (Figures 5A,B), and significant differences were observed (*F* = 13.556, *p* < 0.001 and *F* = 4.088, *p* = 0.02; Figure 5B). Mice in the APP/PS1+NaCl group exhibited higher hippocampal GSK3β levels and lower p-Ser9-GSK3β levels than mice in the Ctrl + NaCl group (*p* < 0.001 and *p* = 0.004; Figure 5B). After the FLX treatment, mice in the APP/PS1+FLX group presented lower hippocampal GSK3β levels and higher p-Ser9-GSK3β levels than mice in the APP/PS1+NaCl group (*p* < 0.001 and *p* = 0.037; Figure 5B); however, the differences in the hippocampal levels of the GSK3β and p-Ser9-GSK3β proteins between the Ctrl + NaCl and Ctrl + FLX groups were not significant (*p* = 0.591 and *p* = 0.641; Figure 5B).

In addition, we semiquantitatively analyzed the hippocampal levels of the 5-HT1AR protein in the four groups (Figures 5A,B), and no significant differences were observed (*F* = 0.619, *p* = 0.611). We next analyzed the numbers of oligodendrocyte precursor cells expressing 5-HT1AR (PFDGαR^+^/5-HT1AR^+^ cells) and mature oligodendrocytes expressing 5-HT1AR (CNPase^+^/5-HT1AR^+^ cells) (Figure 5C) in the hippocampus, once again observing no significant differences among the four groups (Figures 5D,E).

## Discussion

FLX is often used to treat psychiatric symptoms in patients with AD and has been shown to improve their cognition and memory in studies examining the treatment of psychiatric symptoms in patients with AD (Aboukhatwa et al., 2010; Rozzini et al., 2010; Kim et al., 2013). Clinical and basic research studies have shown that FLX delays the occurrence of AD (Ciao et al., 2015; Jin et al., 2017). Here, we administered a low dose (10 mg/kg/d) of FLX intraperitoneally to APP/PS1 transgenic mice with AD for a long period (2 months). FLX delayed deficiencies in the spatial learning and memory, as well as the working and reference memory of mice with AD, without affecting their general attributes, such as body weight, exploratory behaviors and general activities in a novel environment, or their athletic abilities (swimming speed). In recent years, a shift in the treatment window to an early stage was identified as an effective strategy to prevent AD (Crous-Bou et al., 2017; McDade and Bateman, 2017). Here, 8-month-old APP/PS1 transgenic mice were chosen as the study subjects to represent this stage of AD. Reiserer et al. detected Aβ deposition and impaired spatial learning in 7-month-old APP/PS1 mice (Reiserer et al., 2007), and we found observable Aβ levels, Aβ deposition and impaired learning and memory abilities in 10-month-old APP/PS1 mice. In contrast, Minkeviciene et al. did not observe a spatial learning deficit in 10-month-old APP/PS1 mice using the Morris water maze (Minkeviciene et al., 2008), and Garcia-Alloza et al. reported significant increases in soluble and insoluble levels of Aβ40 and Aβ42 at 8 months compared to the levels detected at 4 and 6 months in the APP/PS1 mouse brain; however, 10-month-old APP/PS1 mice presented lower levels of soluble and insoluble Aβ40 and Aβ42 than 8- and 12-month-old mice (Garcia-Alloza et al., 2006). We speculated that the impaired learning and memory abilities of APP/PS1 mice aged between 7 and 10 months might be reversible, similar to changes in Aβ. Thus, we chose to use 8-month-old APP/PS1 transgenic mice without a cognitive impairment or mild cognitive dysfunction to study the preclinical and early stages of AD. In the present study, the FLX intervention delayed the declines in the cognitive abilities of mice with AD during the early stage.

We then sought to examine the mechanism by which FLX prevents the progression of AD. Oligodendrocyte injury and demyelination disrupt brain function (Lee et al., 2012). Desai et al. proposed that early oligodendrocyte/myelin pathology in AD mice was a novel therapeutic target (Desai et al., 2010), and Behrendt et al. reported dynamic myelin aberrations in the white matter of mice and patients with AD (Behrendt et al., 2013). In our previous studies, we observed marked myelinated fiber loss in the hippocampi of Tg2576 and APP/PS1 mice (Lu et al., 2016; Chao et al., 2018). As myelinating glial cells, oligodendrocytes are diffusely distributed within the hippocampus and are required for myelin repair. However, both myelin damage and oligodendrocyte abnormalities are observable in the early stages of AD (Bartzokis, 2004; Destrooper and Karran, 2016; Nasrabady et al., 2018; Zhang et al., 2019). Here, we semiquantitatively analyzed the protein levels of CNPase and MBP in the hippocampus, which are important components of the myelin sheaths and markers of mature oligodendrocytes (Nawaz et al., 2013; Raasakka and Kursula, 2014). Ten-month-old APP/PS1 transgenic AD mice, which displayed decreased learning and memory abilities, presented a CNPase and MBP deficiency in the hippocampus. In addition, we quantified CNPase^+^ cells in the hippocampus and found a loss of CNPase^+^ cells in the hippocampus of 10-month-old APP/PS1 transgenic AD mice. In our previous study, 10-month-old APP/PS1 transgenic AD mice displayed decreases in the total volumes of myelinated fibers and myelin sheaths in the hippocampus (Chao et al., 2018). Thus, the changes in mature oligodendrocytes (CNPase^+^ cells) are consistent with the changes in the myelin sheath in the hippocampi of APP/PS1 transgenic AD mice. Behrendt et al. reported a lower ratio of CNP to axonal neurofilament 70 in 6-month-old APP/PS1 mice than in control mice, whereas this ratio in 9-month-old APP/PS1 mice was similar to control mice (Behrendt et al., 2013). The authors proposed that APPPS1 mice did not exhibit any further age-dependent increase in myelin defects between 6 and 9 months. However, in our study, we observed marked losses of mature oligodendrocytes and myelin sheaths in the hippocampi of 10-month-old APP/PS1 mice. Three factors may potentially explain the difference. (1). The analysis of different ages: Behrendt et al. studied the myelin sheaths of APP/PS1 mice at 6 and 9 months of age (Behrendt et al., 2013), whereas we studied the myelin sheaths of APP/PS1 mice at 10 months of age. As noted above, dynamic changes occur in both Aβ levels and the learning abilities of APP/PS1 mice at <10 months of age, and dynamic changes in myelin and oligodendrocytes must thus also occur. (2). The analysis of different regions: Behrendt et al. analyzed myelin throughout the brain (Behrendt et al., 2013), whereas we analyzed myelin in the hippocampus. In our previous studies, we reported differential changes in the white matter and hippocampus, as myelin loss occurred in the hippocampus rather than in the white matter of APP/PS1 mice at 10 months of age (Zhang et al., 2017; Chao et al., 2018). However, in 10-month-old APP/PS1 mice, damage to the myelin sheath and mature oligodendrocytes was not ameliorated, at least in the hippocampus.

Two possible factors might underlie the loss of mature oligodendrocytes in the hippocampus of APP/PS1 transgenic mice: oligodendrocyte development dysfunction and mature oligodendrocyte death. A previous study reported the upregulation of neuregulin-1 type III, a critical protein for oligodendrocyte development, in the hippocampi of 2-month-old APP/PS1 mice (Wu et al., 2017). The development of oligodendrocytes involves several processes: proliferation, differentiation and maturation. NG2 is a marker that reflects the proliferation of oligodendrocytes (Dawson et al., 2003). In this study, we semiquantitatively analyzed the level of the NG2 protein in the hippocampus, and observed a similar NG2 level in the hippocampus in 10-month-old APP/PS1 mice and control mice. The chondroitin sulfate proteoglycan NG2 is expressed on NG2^+^ cells, and NG2^+^ cells can downregulate NG2 and differentiate into oligodendrocytes (Dimou and Gallo, 2015). However, a clinical study has shown that patients clinically diagnosed with AD present significantly decreased levels of soluble NG2 in the cerebrospinal fluid compared with individuals without dementia (Nielsen et al., 2013). In contrast, Behrendt et al. reported a higher density of newborn NG2 cells in the cortical gray matter and white matter of APP/PS1 mice at 6 months of age (Behrendt et al., 2013). The proliferation of oligodendrocytes appears to be altered in individuals with AD, but a difference in the changes in oligodendrocytes has been observed between AD animal models and patients with AD. We labeled cells of the oligodendrocyte lineage with an antibody against Olig2, which is the key to fate determination and the early differentiation of the oligodendrocyte lineage, to identify changes in oligodendrocyte differentiation. Olig2 knockout mice do not produce oligodendrocytes in the CNS, and Olig2 expression gradually decreases during the period of maturity until it is no longer expressed (Lu et al., 2002; Li et al., 2011). We counted the total number of Olig2^+^ cells in the hippocampi of AD mice and found that 10-month-old APP/PS1 mice exhibited an abnormal increase in the number of Olig2^+^ cells in the hippocampus. Similarly, Behrendt et al. studied the changes in Olig2^+^ cells in AD and found a higher density of Olig2^+^ cells in the cortical gray matter of 6-month-old APPPS1 mice (Behrendt et al., 2013). Conversely, Behrendt et al. reported a lower density of Olig2^+^ cells in the cortical gray matter and white matter in postmortem samples from humans with AD (2013). The changes in oligodendrocyte differentiation appear to differ between AD animal models and patients with AD. Animal models of AD usually simulate patients with mild to moderate AD, e.g., APP/PS1 transgenic AD mice (Bilkei-Gorzo, 2014). As mentioned above, the 8-month-old APP/PS1 transgenic mice we chose represent only the preclinical or early stage of AD. However, patients with AD are already in later stages of the disease upon clinical diagnosis. In the study by Behrendt et al., the postmortem samples were obtained from humans with AD between 68 and 85 years of age (Behrendt et al., 2013). Similarly, in the study by Nielsen et al. the subjects were patients with AD between 64 and 87 years of age (Nielsen et al., 2013). We speculated that oligodendrocyte development might be increased in the early stages of AD, whereas it might be decreased in the late stages of AD. Early oligodendrocyte pathology in AD might be a key factor in the prevention and treatment of AD.

Since hippocampal oligodendrocyte development is increased in 10-month-old APP/PS1 mice, why are fewer mature oligodendrocytes still detected in their hippocampi? Therefore, we analyzed the ratio of mature oligodendrocytes to oligodendrocyte lineage cells in the hippocampi of 10-month-old APP/PS1 mice and found that the APP/PS1 mice presented more immature oligodendrocytes but fewer mature oligodendrocytes in the hippocampus than their non-transgenic littermates. Then, we evaluated the newborn oligodendrocyte lineage cells (BrdU^+^/Olig2^+^) in the hippocampi of 10-month-old APP/PS1 mice, but did not observe significant differences in the densities of BrdU^+^/Olig2^+^ cells in the hippocampal subregions between the AD mice and the non-transgenic littermates. Behrendt et al. reported an increase in the number of BrdU^+^/Olig2^+^ cells in the cortical gray matter of APP/PS1 mice at 6 and 11 months of age (Behrendt et al., 2013), whereas Kamphuis et al. reported no change in the number of BrdU^+^/Olig2^+^ cells in the cortex of APP/PS1 mice at 3, 6, 9, 12, and 15 months of age (Kamphuis et al., 2012). Although we were unable to directly compare our current results with their findings, the issue of the newborn oligodendrocyte lineage remains controversial. In the current study, we first observed an increase in the number of newborn Olig2^+^ cells in the hippocampi of APP/PS1 mice at 10 months of age. Then, we evaluated the maturation of newborn Olig2^+^ cells in the hippocampi of 10-month-old APP/PS1 mice and found a significantly lower density of newborn mature oligodendrocytes and their ratio to newborn oligodendrocyte lineage cells in the DG of AD mice than in the non-transgenic littermates. The newborn Olig2^+^ cells in the DG of AD mice likely failed to mature. We analyzed the density of SOX10^+^ cells in the hippocampus to further clarify whether dysmaturity existed in the oligodendrocytes and observed significantly lower densities of SOX10^+^ cells in the CA2-3 fields and DG of 10-month-old APP/PS1 mice than in non-transgenic littermate mice. SOX10 is expressed in oligodendrocytes, and a previous study showed that oligodendrocyte progenitor cells develop normally in the absence of the SOX10 gene, but did not differentiate into mature oligodendrocytes in the mouse spinal cord in a timely manner (Stolt et al., 2002). As a universal regulator of myelin gene expression, SOX10 regulates the expression of various myelin-related genes at the transcriptional level by binding to the promoters of myelin-related genes, such as protein-lipid protein (PLP) and MBP (Schreiner et al., 2007; Turnescu et al., 2018). The expression of SOX10 may be gradually decreased during the maturation of oligodendrocytes in the CA2-3 fields and DG, which might be an important cause of Olig2^+^ cell maturation failure in the CA2-3 fields and DG of 10-month-old APP/PS1 mice. In the CA1 field, we did not obtain evidence that the loss of mature oligodendrocytes is related to the failure of Olig2^+^ cell maturation and SOX10 levels, and the cause of oligodendrocyte loss in this field remains to be studied.

In addition to dysfunctional oligodendrocyte development, the death of mature oligodendrocytes might also induce a decrease in the number of mature oligodendrocytes. In this study, we detected higher levels of Aβ40 and Aβ42 and larger amyloid plaques in the hippocampi of 10-month-old APP/PS1 mice. Jantaratnotai et al. reported that Aβ induces oligodendrocyte death in the rat brain after a stereotaxic injection of Aβ1-42 into the brain (Jantaratnotai et al., 2003). As shown in the study by Xu et al., Abeta 1-40 and a truncated fragment, Abeta 25-35, induce the death of oligodendrocytes expressing intermediate differentiation markers *in vitro* (Xu et al., 2001). Lee et al. reported that Aβ25-35 induces the death of mature oligodendrocytes (Lee et al., 2004). Nielsen et al. reported decreased NG2 concentrations in both cell lysates and supernatants when oligodendroid precursor cells (NG2 cells) were exposed to fibrillar Aβ1-42 *in vitro*, and both oligomeric and fibrillar Aβ1-42 induced changes in NG2 cell morphology (Nielsen et al., 2013). In addition, β-amyloid induced p16 and p21 protein expression in oligodendrocyte progenitor cells (Olig2 and NG2 cells) and then induced oligodendrocyte progenitor cell senescence (Zhang et al., 2019). In the present study, the ratio of oligodendrocytes (Olig2^+^ cells) expressing p16 was higher in the hippocampi of 10-month-old APP/PS1 mice, and this change in the hippocampus was consistent with the loss of mature oligodendrocytes. We speculated that β-amyloid not only causes the death of mature oligodendrocytes but also causes the senescence of oligodendrocyte precursor cells, thus causing dysfunction in oligodendrocyte maturation and decreasing the number of mature oligodendrocytes. Regardless, both dysfunction in oligodendrocyte development and the death of mature oligodendrocytes induced a decrease in the number of mature oligodendrocytes. Our results provide supporting evidence for changes in oligodendrocytes in the hippocampus during early AD, which might be the important pathological structural basis and therapeutic target of AD.

While hippocampal oligodendrocyte abnormalities are observed in mice with early-stage AD, the ability of the FLX treatment to reverse these oligodendrocyte abnormalities remains an open question. In the present study, FLX had no effect on the levels of MBP and CNPase in the hippocampus but decreased the density of mature oligodendrocytes (Olig2^+^/CNPase^+^) within the CA1 field in normal mice. However, the FLX treatment induced the expression of oligodendrocyte-related proteins, such as MBP and CNPase, in the hippocampus and not only rescued the decrease in the total number of mature oligodendrocytes but also reversed the abnormal increase in the total number of oligodendrocyte lineage cells within the CA1 field, CA2-3 fields and DG of APP/PS1 transgenic mice. Notably, the FLX intervention did not affect the total number of mature oligodendrocytes in the normal mouse hippocampus, as reported in normal rats (Kroeze et al., 2015); however, it significantly decreased the ratio of mature oligodendrocytes to oligodendrocyte lineage cells within the CA1 field of the hippocampus in normal mice. Marcussen et al. reported that intraperitoneal injections of FLX at a low dose and for a long period increased neurogenesis in Wistar rats (Marcussen et al., 2008). Furthermore, de Leeuw et al. found that FLX upregulated oligodendrocyte markers in neural embryonic stem cells, as revealed by a cell lineage map (de Leeuw et al., 2020), indicating that FLX might promote oligodendrogenesis under normal conditions. Regarding the lack of effect of FLX on mature oligodendrocytes, FLX promotes oligodendrogenesis, which may lead to a decreased proportion of mature oligodendrocytes. The proportion of mature oligodendrocytes was only decreased in the CA1 field and may be related to the special function of the hippocampal CA1 region, but this topic requires further study. The FLX intervention did not increase the number of mature oligodendrocytes in the hippocampus of normal mice, whereas the FLX intervention exerted a protective effect on mature oligodendrocytes in the hippocampus of APP/PS1 mice. FLX has been shown to prevent the amelioration of myelination deficits in mice with multiple system atrophy (Ubhi et al., 2012). Moreover, the FLX treatment increases oligodendrocyte components in depressed rodent models (Kodama et al., 2004; Surget et al., 2009). In our previous study, FLX increased the total number of CNPase^+^ cells in the hippocampi of rats with chronic unpredictable stress-induced depression (Wang et al., 2020). Thus, FLX might protect oligodendrocytes in a state of illness. However, no previous studies have examined the effect of FLX on oligodendrocytes in individuals with AD. In the present study, we studied this effect for the first time and found that FLX rescued the decrease in the number of mature oligodendrocytes, reversed the abnormal increase in the number of oligodendrocyte lineage cells in the hippocampi of AD mice, and then promoted the maturation of oligodendrocytes in the hippocampi of AD mice. First, the FLX treatment not only increased the density of newborn mature oligodendrocyte cells in the CA2-3 fields of normal mice but also increased the ratios of newborn mature oligodendrocytes to newborn oligodendrocyte lineage cells within the CA2-3 field and DG of APP/PS1 transgenic mice, indicating that FLX might promote the maturation of newborn oligodendrocytes. As mentioned above, the FLX intervention might promote oligodendrogenesis in the CA1 field of normal mice. We speculated that FLX not only promotes oligodendrogenesis in the CA2 and CA3 fields but also promotes oligodendrocyte maturation. Regarding the simultaneous increases in the numbers of Olig2^+^ and CNPase^+^ cells, the proportion of mature oligodendrocytes was not affected in CA fields 2 and 3. Oligodendrocytes metabolically support axon survival and function through mechanisms independent of myelination (Lee et al., 2012). We speculate that the neurons and axons in the CA1, CA2, and CA3 fields may have different metabolic requirements and thus have different requirements for oligodendrogenesis, but this phenomenon requires further study. Furthermore, FLX increased the number of SOX10^+^ cells within the CA2-3 fields and DG and the number of SOX10^+^/CNPase^+^ cells within the CA2-3 fields of AD mice, suggesting that FLX might promote SOX10 expression on oligodendrocytes during the maturation of oligodendrocytes in the CA2-3 fields and the subsequent differentiation of mature oligodendrocytes. Our results appear to provide evidence for the effects of FLX on the development of oligodendrocytes in the hippocampus of a mouse AD model. Although we were unable to obtain support for this effect of FLX on oligodendrocytes from previous studies, we obtained clues from the relative effects of FLX on normal mice. Kodama et al. reported that FLX increased cell proliferation in the hippocampus and prefrontal cortex of adult rats (Kodama et al., 2004). In particular, Encinas et al. reported that FLX increased the number of early progenitor cells in the adult hippocampus (Encinas et al., 2006). In addition to promoting oligodendrocyte development, FLX might reduce the death of mature oligodendrocytes. FLX might prevent oligodendrocyte cell death after spinal cord injury (Lee et al., 2015). In our study, after FLX exposure, lower levels of Aβ40, Aβ42, and amyloid plaque formation were observed in the hippocampi of the FLX-treated transgenic AD mice than in the hippocampi of transgenic AD mice administered normal saline, consistent with previous studies (Wang et al., 2014; Ciao et al., 2015). Moreover, FLX exposure decreased the ratio of oligodendrocytes expressing p16 in the hippocampi of AD mice. The expression of p16 in oligodendrocytes indicates the senescence of oligodendrocytes (Zhang et al., 2019), and senescent immature oligodendrocytes might lose the ability to mature. We speculated that FLX inhibited the expression of p16 in oligodendrocytes and then prevent the senescence of oligodendrocytes, thereby promoting the maturation of oligodendrocytes in the hippocampi of AD mice. FLX might protect mature oligodendrocytes by reducing the death of mature oligodendrocytes and promoting the development of oligodendrocytes.

Regarding the mechanism by which FLX affects hippocampal oligodendrocytes in APP/PS1 mice, as an SSRI antidepressant, FLX mainly functions by increasing the concentration of extracellular 5-HT through the inhibition of 5-HT reuptake by synapses, and the effect of 5-HT depends on its binding to its receptor (Dringenberg and Diavolitsis, 2002; Perez-Caballero et al., 2014). FLX activates 5-HT1ARs in the brains of mouse models of emotional impairments and schizophrenia by increasing the 5-HT concentration, thereby inhibiting the expression of GSK3 (Li et al., 2007; Haroutunian et al., 2014). GSK3β, a subtype of GSK3, is an important negative regulator of oligodendrocyte differentiation and myelination (Azim and Butt, 2011). In the present study, no significant differences in hippocampal 5-HT1AR protein expression were observed between the transgenic mice with AD and the non-transgenic littermate mice or between the mice in the normal saline and FLX groups. Based on our results, the expression of 5-HT1AR was not decreased in the hippocampi of mice with AD, and FLX had no effect on 5-HT1AR expression in the hippocampus of the mouse AD model. We also analyzed the numbers of oligodendrocyte precursor cells and mature oligodendrocytes expressing 5-HT1AR in the hippocampus, and no significant differences were observed among the four groups. However, the GSK3β protein was expressed at significantly higher levels in the hippocampus of transgenic APP/PS1 mice than in the hippocampi of non-transgenic littermate mice, while the hippocampal level of GSK3β phosphorylated at Ser9 was significantly lower in the transgenic AD mouse model than in the hippocampi of non-transgenic littermate mice. Compared to the normal saline treatment, the FLX treatment decreased the expression of the GSK3β protein and increased the level of GSK3β phosphorylated at Ser9 in the hippocampi of AD mice. GSK-3β is a serine (Ser)- and threonine (Tyr)-rich kinase that is activated by the phosphorylation of Tyr216 residues and inhibited by the phosphorylation of Ser9 residues (Frame et al., 2001). The increased level of the GSK3β protein phosphorylated at Ser9 indicates the inhibition of its activity. Based on our results, FLX inhibited GSK3β expression and activity in the hippocampi of AD mice. GSK3β inhibition not only increases the number of oligodendrocytes and oligodendrocyte precursor cells but also promotes the formation of myelin (Azim and Butt, 2011). FLX might promote oligodendrocyte maturation by inhibiting the expression and activity of GSK3β in the hippocampus of the mouse AD model. In other words, FLX inhibits the expression and activity of the GSK3β protein, but not by increasing the levels of 5-HT1AR. Numerous other mechanisms might affect GSK3β, such as the antagonization of LINGO1 to inhibit GSK3 activity (Zhao et al., 2008), but these mechanisms require further research.

## Conclusions

Early oligodendrocyte pathology in AD might be a key factor in the prevention and treatment of AD. FLX may delay the senescence of oligodendrocyte lineage cells and promote oligodendrocyte maturation in the hippocampus of the APP/PS1 transgenic mouse. In addition, FLX may regulate GSK3β through a mechanism other than 5-HT1AR and then inhibit the negative effect of GSK3β on oligodendrocyte maturation. However, its further mechanisms remain to be studied. Nevertheless, FLX might be a safe and effective medication for preventing or delaying the progression of AD by ameliorating early oligodendrocyte pathology, which might be regarded as a novel therapeutic target.

## Data Availability Statement

The original contributions presented in the study are included in the article/supplementary materials, further inquiries can be directed to the corresponding author/s.

## Ethics Statement

The animal study was reviewed and approved by the Animal Care and Research Committee of Chongqing Medical University.

## Author Contributions

F-lC, YZ, and YT: conception and design of the study. F-lC and YZ: acquisition and analysis of data. F-lC, YZ, LZ, C-nZ, XL, JT, J-hF, X-yD, and YT: drafting of a significant portion of the manuscript or figures. All authors contributed to the article and approved the submitted version.

## Conflict of Interest

The authors declare that the research was conducted in the absence of any commercial or financial relationships that could be construed as a potential conflict of interest.
